# A multi-center, pragmatic, effectiveness-implementation (hybrid I) cluster randomized controlled trial to evaluate a child-oriented goal-setting approach in paediatric rehabilitation (the ENGAGE approach): a study protocol

**DOI:** 10.1186/s12887-022-03381-4

**Published:** 2022-06-29

**Authors:** Lesley Pritchard-Wiart, Sandy Thompson-Hodgetts, Ashley B. McKillop, Rhonda Rosychuk, Kelly Mrklas, Lonnie Zwaigenbaum, Jennifer Zwicker, John Andersen, Gillian King, Pegah Firouzeh

**Affiliations:** 1 Department of Physical Therapy, Faculty of Rehabilitation Medicine, University of Alberta, University of Alberta, 3-60 Corbett Hall, Edmonton, AB T6G 2G4 Canada; 2 Department of Occupational Therapy, Faculty of Rehabilitation Medicine, University of Alberta, 3-20 Corbett Hall, Edmonton, AB T6G 2G4 Canada; 3 Faculty of Rehabilitation Medicine, University of Alberta, University of Alberta, 3-78 Corbett Hall, Edmonton, AB T6G 2G4 Canada; 4 Department of Pediatrics, Faculty of Medicine and Dentistry, University of Alberta, 3-524 Edmonton Clinic Health Academy, 11405 87 Ave, Edmonton, AB T6G 1C9 Canada; 5 System Innovation and Programs, Alberta Health Services and Department of Community Health Sciences, Cumming School of Medicine, University of Calgary, Strategic Clinical Networks, Provincial Clinical Excellence, Alberta Health Services and Department of Community Health Sciences, Cumming School of Medicine, University of Calgary, Calgary, Alberta Canada 1403 29th St NW, T2N 2T9; 6 Department of Pediatrics, Faculty of Medicine and Dentistry, University of Alberta, 10230 111 Ave NW, Edmonton, AB T5G 0B7 Canada; 7 Director School of Public Policy, Cumming School of Medicine, University of Calgary, NW University of Calgary, 135 376 Collegiate Blvd 2500 University Drive, NW, Calgary, AB T2N 1N4 Canada; 8 Holland Bloorview Kids Rehabilitation Hospital, 150 Kilgour Rd, East York, ON M4G 1R8 Canada; 9 Faculty of Rehabilitation Medicine, University of Alberta, University of Alberta, 3-70 Corbett Hall, Edmonton, T6G 2G4 Canada

**Keywords:** Pediatrics, Rehabilitation, Pragmatic trial, Goal-setting, Implementation evaluation

## Abstract

**Background:**

Child-oriented goal-setting in pediatric rehabilitation may improve child motivation, engagement in therapy, child outcomes related to therapy, and service delivery efficiency. The primary objective of this trial is to determine the effectiveness of a principles-driven, child-focused approach to goal-setting, Enhancing Child Engagement in Goal-Setting (ENGAGE), on pediatric rehabilitation outcomes compared to usual practice. The three secondary objectives are to 1) compare costs and secondary outcomes of the ENGAGE approach to usual practice, 2) determine the influence of child, parent and therapist characteristics on child engagement in therapy and rehabilitation outcomes, and 3) identify barriers and facilitators to the implementation of ENGAGE.

**Methods:**

This research protocol describes a pragmatic, multi-site, cluster, effectiveness-implementation (hybrid type 1 design) randomized controlled trial. Therapists (*n* = 12 clusters of two therapists) at participating sites (*n* = 6) will be randomized to 1) the ENGAGE intervention group, or 2) usual care (control) using a computer-generated, permuted-block randomization sequence with site as a stratification variable designed by a statistician (RR). Each therapist will recruit four children 5–12 years old with neurodevelopmental conditions (*n* = 96), who will receive ENGAGE or usual care, according to therapist group allocation. ENGAGE therapists will be trained to use a 'toolbox' of evidence-driven, theory-informed principles to optimize child and parent motivation, engagement in the goal-setting process, and performance feedback strategies. Outcomes include goal performance (primary outcome), engagement in therapy, functional abilities, participation, and parent and child quality of life. Qualitative interviews with children, parents, ENGAGE therapists, and managers will explore challenges to implementation and potential mitigation strategies. Mixed effects multiple linear regression models will be developed for each outcome to assess group differences adjusted for clustering. A cost-effectiveness analysis will combine cost and a measure of effectiveness into an incremental cost-effectiveness ratio. Qualitative data on implementation will be analyzed inductively (thematic analysis) and deductively using established implementation science frameworks.

**Discussion:**

This study will evaluate the effects of collaborative goal-setting in pediatric rehabilitation and inform effective implementation of child-focused goal-setting practices.

**Trial Registration:**

NCT05017363 (registered August 23, 2021 on ClinicalTrials.gov).

## Background

Many children with neurodevelopmental conditions access rehabilitation services, such as occupational therapy (OT) and physical therapy (PT), to optimize functional abilities and meaningful participation and inclusion in important life activities [1]. Thus, a focus on individualized goal setting and identifying outcomes that are meaningful to children and families is a foundational component of OT and PT intervention [2, 3]. Despite the importance of focusing therapy on activities that are meaningful to children and families, implementing individualized goal-setting in pediatric rehabilitation has been challenging [4–7]. Our previous research [6, 8–11] and work by others [3, 4, 12, 13] indicates that parent engagement in goal setting processes is suboptimal for various reasons including (1) mismatch between clinician and parent perspectives on appropriate goals, (2) lack of therapist confidence in the abilities of parents to identify meaningful goals, and (3) organizational barriers such as lack of time, challenges with service team coordination, and poor documentation. Even less emphasis is placed on ensuring children are optimally engaged in the goal setting process [13], despite evidence that perceived goal importance plays a crucial role in behavior change that leads to goal performance [14]. For example, some therapists worry that engaging a child with autism in goal-setting may perpetuate restricted interests; while parents and youth with autism perceive goals to be more meaningful when they are grounded in activities the child finds engaging [10].

Collaborative goal-setting aligns therapy with individualized goals of children and families; enacting the tenets of family-centered care. This approach is recognized as best practice with children with disabilities [12, 15]. Furthermore, engaging children in goal-setting could positively affect rehabilitation outcomes due to increased motivation to participate in goal-related therapy activities [13, 16], particularly since children as young as five are capable of identifying achievable goals [17, 18]. Collaborative goal-setting may also improve service delivery efficiency by providing more targeted interventions [4].

**Fig. 1:**
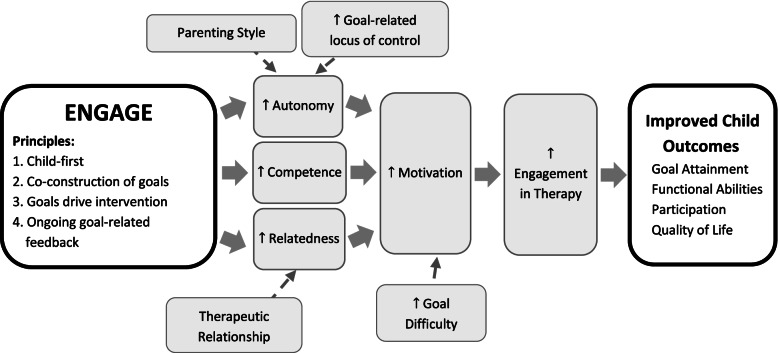
Study conceptual model (moderators indicated with thin arrows)

To evaluate the effects of child-focused goal setting on outcomes and service delivery efficiency, we have developed the Enhancing Child Engagement in Goal Setting (ENGAGE) approach**.** ENGAGE operationalizes principles of relevant theoretical frameworks [13, 19], contemporary approaches to rehabilitation emphasizing individualized goals [20, 21], and the evidence supporting children’s ability to engage in goal setting [18]. ENGAGE aims to ensure that children have a voice by optimizing their involvement in goal-setting and encouraging clinical judgment in tailoring the use of tools and strategies to children and families’ needs. There is evidence that prescriptive, one-size-fits-all approaches have resulted in disappointing uptake elsewhere [3]. In addition, variability in parenting styles, such as the extent to which a parent is comfortable with adult versus child-directed decision-making, should be considered in child-focused approaches to rehabilitation goal-setting. See Fig. 1 for the ENGAGE study theoretical framework.

It is well established that integrating new, evidence-informed approaches, including goal-setting approaches, into daily practice is incredibly challenging [22]. Process, intervention characteristics, people influences, and context of an intervention, rather than evidence for its effectiveness, can play a more critical role in its successful adoption and widespread use [23]. Therefore, an important component of facilitating uptake of a new approach is identifying and understanding barriers and facilitators to implementation to guide the development of targeted implementation strategies [24–26]. Implementation evaluations are crucial for closing research to practice gaps and have influenced significant practice changes in other areas of health care [27].

The primary clinical effectiveness trial objective is to determine the effectiveness of a collaborative goal-setting approach on therapists’ perception of child engagement in therapy, goal performance (primary outcome), functional abilities, participation in home, school and/or community, and child and caregiver quality of life compared to usual practice. The secondary effectiveness evaluation objectives are: 1) to assess the incremental cost of ENGAGE compared to usual care relative to the study outcomes, and 2) to determine the influence of child, parent, and therapist characteristics on child engagement and outcomes.

The implementation evaluation objectives are: 1) to identify barriers and facilitators to implementation of ENGAGE, such as clinical contexts, child and family characteristics, and specific features of ENGAGE, 2) to understand family and therapist perspectives on the key components of ENGAGE that are associated with perceived effectiveness, and 3) to determine if differences are present in barriers and facilitators to implementation in rural and urban pediatric rehabilitation sites, and in different types of programs.

## Methods/design

### Study setting

The study will be conducted in six established pediatric rehabilitation sites in rural and urban settings in Alberta, Canada. Goal-setting and intervention can occur in a variety of settings (e.g., clinic, family home). The study opened to enrolment in February 2022.

### Trial design

This is a pragmatic, cluster randomized controlled trial (RCT) with therapists randomized to one of two groups; the ENGAGE intervention group or the usual care control group. The RCT will be a six-site trial with two groups (ENGAGE training absent/present) with two periods of post-intervention assessment (immediate post-treatment and three-month follow-up). Core features of a pragmatic design include a comparison of clinically feasible and relevant interventions, the inclusion of diverse patient populations and practice settings, and the measurement of a broad range of outcomes [28]. This trial can also be categorized as a Hybrid Type 1 design [29], a clinical intervention coupled with observing implementation with intent to inform scale and spread. The methodology specific to the clinical effectiveness trial will be described separately from the implementation evaluation. A cost-effectiveness analysis will also be conducted in conjunction with the RCT.

### Clinical effectiveness trial methodology

#### Eligibility criteria

Consistent with eligibility determination recommended for pragmatic trials [30], inclusion criteria are broad and exclusion criteria are minimal. Inclusion criteria are children who: 1) are 5–12 years old, 2) are referred to PT and/or OT for a period of direct treatment, 3) are able to engage in the goal-setting process by communicating verbally or non-verbally (based on therapist clinical judgment), and 4) understand English. Children will be excluded from the trial if 1) the parent or guardian who attends therapy does not speak English, 2) the child has a diagnosis associated with developmental/neurological regression, such as uncontrolled seizures.

### Sample size

The sample size will be 96 children (12 therapists as clusters per group and four children per therapist) recruited from the six separate sites by the study therapists (4 children/therapist). Based on our pilot work, we anticipate that child dropout from the pre-post intervention period will be minimal (i.e., less than 5%). A target change score of 2.0, a clinically significant change on the Canadian Occupational Performance Measure (goal performance rating) (COPM-P; primary outcome) [31], with a standard deviation of 2.75 [32] corresponds to an effect size of 0.723 for the comparison of means. A sample size of 96 will enable us to detect an effect size of at least 0.682 in the primary outcome (alpha = 0.05 and 80% power), assuming an intra-cluster correlation (ICC) of 0.1 using a 2-sided, cluster-adjusted, t-test for the comparison of means. We selected an ICC of 0.1 based on the results of a previous cluster RCT with children with cerebral palsy (ICCs between 0.08 to 0.13) [32]. Since therapist attrition is possible over the duration of the study, a cluster size of 11 would still provide 80% power to detect an effect size of 0.716, below our target effect size. Smaller effect sizes will be detectable if the ICC is smaller than 0.1.

### Consent and confidentiality

All parents will sign an informed consent document and children nine years of age and older will sign an assent form. Forms will be provided to the families by study therapists and returned directly to the study investigators. All data will be stored separately from identifiers on a password-protected, secure server at the University of Alberta. Only the list authors will have access to the trial dataset.

### Randomization

**Fig. 2:**
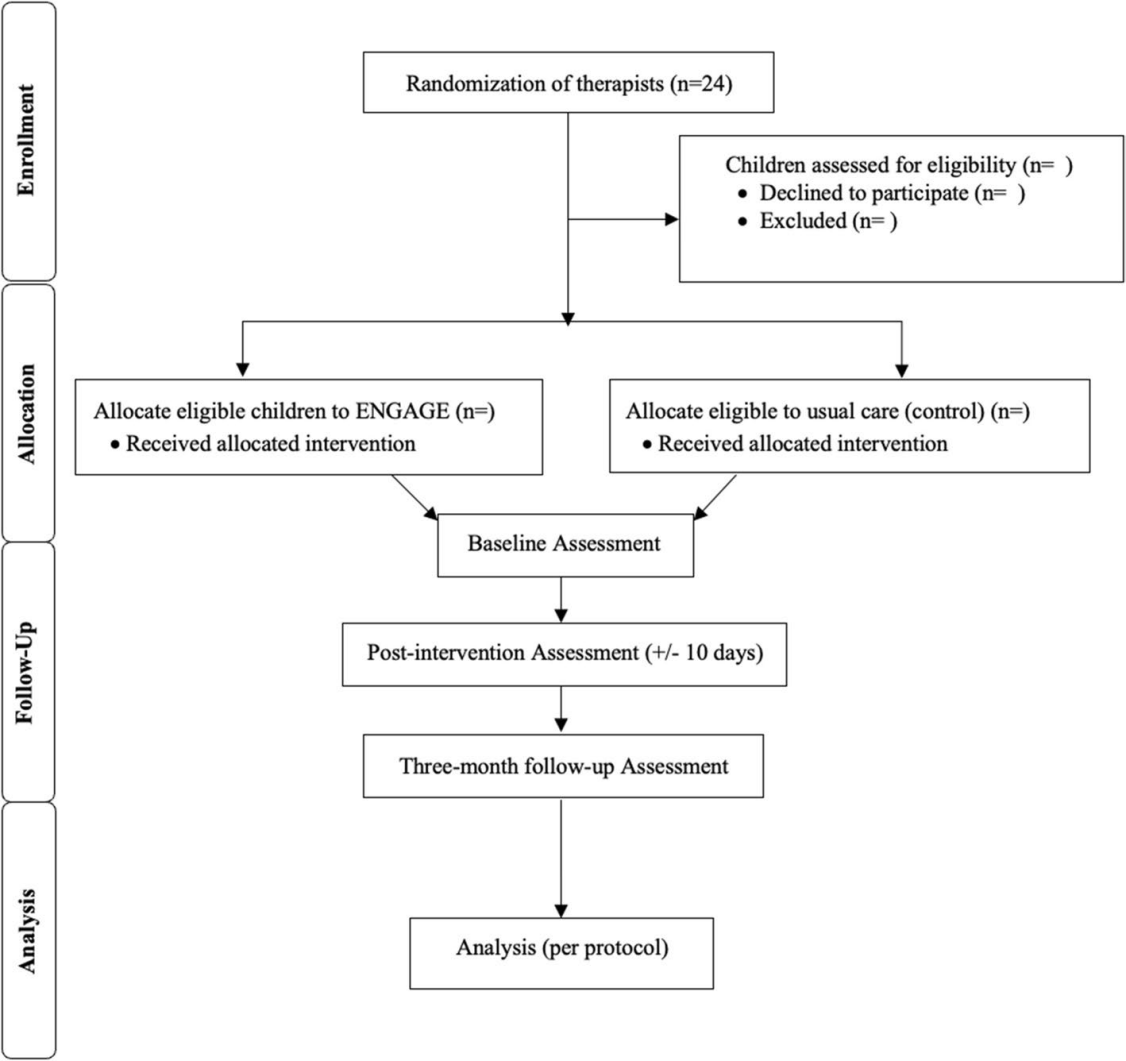
CONSORT 2010 flow diagram

A computer-generated, permuted-block randomization sequence stratified by site will be used to allocate 24 OTs and PTs across six sites to ENGAGE or usual care to ensure balanced groups. Blocked randomization by site will facilitate consistency of therapy interventions and child characteristics between groups with the exception of ENGAGE in the intervention. If therapists within more than one program per site participate, therapists will also be randomized by program. The randomization sequence will be uploaded to Research Electronic Data Capture (REDCap) [33] to allow centralized, online randomization. Randomization of therapists (and not children) will decrease contamination in the usual practice group (i.e., so therapists are not asked to go between intervention and usual care practices). Blocked randomization by site will facilitate equal group distribution related to therapy interventions (e.g., types of therapy) and child characteristics (e.g., age, diagnosis). See Fig. 2 for the Consolidated Standards of Reporting Trials (CONSORT) study flowchart.

### Blinding

Randomization will be conducted by a research assistant unfamiliar with therapist practices and who does not have access to the random allocation template. Trained assessors will complete all pre-, post-, and follow-up assessments and will be unaware of therapist and child group allocation. Assessors and family members will be masked to all previous responses on the measures during the post-intervention and follow-up assessments. Data analysis will be conducted by the statistician, masked to group allocation. Blinding of child and family to group allocation is not possible given the nature of the intervention.

### Treatments

**Fig. 3:**
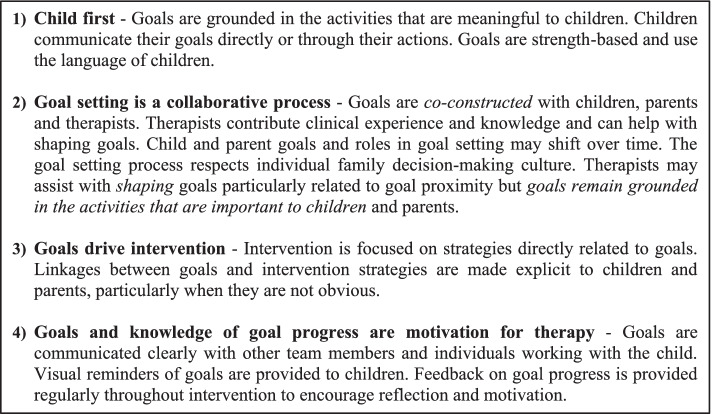
Guiding principles of ENGAGE

*ENGAGE* Therapists will receive manualized training on the ENGAGE principles (see Fig. 3), and child-engagement and goal-setting strategies provided in the ENGAGE toolbox. Training will be led by two experienced clinicians and ENGAGE developers based on a standardized manual and training procedure. Training will include an overview of the Perceived Efficacy and Goal Setting Tool (PEGS) [17], an established goal-setting tool for children aged 5–9. In addition, we will provide training on administration of the COPM [31] with children, the most widely used goal-setting tool in pediatric rehabilitation [34], used with children as young as seven years [31]. We will also introduce strategies used successfully in our pilot and foundational work [35, 36] to assist with goal identification. During the training, we will have ongoing discussions with therapists to identify additional strategies or tools that align with the principles of ENGAGE that could be used across the sites.

With the exception of incorporating the ENGAGE principles, which include goal setting, goal-related feedback, and focus on goal-directed intervention strategies, rehabilitation interventions used to achieve identified goals will not vary from usual practice. Consistent with a pragmatic trial, this approach will enable us to evaluate the effectiveness of ENGAGE in typical clinical settings. Because therapists will be paired within programs, differences in interventions provided and the population served are minimized. This will increase the likelihood that treatment and child characteristics, other than the goal-setting intervention, will be similar between the ENGAGE and usual care groups. Treatment duration and intensity will differ based on various factors including the nature of goals, therapist approach, treatment strategies used, and family preferences, which is consistent with clinical practice. It is anticipated that treatment block lengths will vary from 3–8 sessions over 4–12 weeks, representing typical variation in clinical practice.

*Usual Care (Control)* Control therapists will provide the investigators with goals, but they will not receive any training on child engagement strategies for goal-setting or feedback on how/with whom goals are established. Our pilot study confirmed that adherence to the four ENGAGE principles was a significant shift from traditional practice that does not emphasize child engagement, attention to self-efficacy, or strategies based on principles of behavior change [36]. Based on previous research [34], pilot results [36] and our understanding of clinical practice, we anticipate that the control therapists may engage parents to varying extents in goal identification but will not be using strategies to maximize engagement of the child. To decrease contamination risk, we will emphasize the importance of not sharing strategies or discussing principles with study therapists in the usual care group, and we will monitor and compare practices between the two groups on an ongoing basis.

### Fidelity monitoring

Following the training, we will track the strategies used by both groups of therapists to evaluate treatment fidelity prior to recruiting participants to ensure 1) that therapists in the ENGAGE group are adhering to intervention principles, and 2) that the practices in the two groups are different. Recruitment and formalized data collection will begin once intervention-group therapists at the site achieve an acceptable level of fidelity defined as adherence to ENGAGE principles and group practices are established to be different, or repeated attempts to support therapist in using the ENGAGE approach have been exhausted. Intervention-group therapists will self-report their adherence to ENGAGE principles using a Likert scale. For example, they will report on the extent to which children are involved in identifying their own goals and their use of feedback on goal-related performance at each treatment session. Practices in the control group will be tracked using a form with open-ended questions to prevent contamination from exposure to ENGAGE principles. Ongoing documentation of practices and monitoring will be used to evaluate the need for additional or different implementation support in the intervention group. Co-interventions will be monitored bi-weekly.

### Outcomes

Assessments will be conducted at three time points: 1) baseline (pre-treatment), 2) post-intervention (± 10 days) (primary endpoint), and 3) three months post-intervention to evaluate longer-term effects of ENGAGE. All outcomes data will be collected using electronic forms and transferred to RedCap. Data will be reviewed and verified by an independent research assistant.

### Primary outcome

The primary outcome is self-rated goal performance on the COPM (COPM-P) [31]. The COPM-P was selected as the primary outcome because we believe that attainment of outcomes meaningful to the individual is the most important outcome of therapy. Furthermore, the diversity of intervention approaches and goals in this study necessitated the use of a general, individualized assessment tool. ENGAGE therapists will identify goals with children and can use the COPM goal-setting process if they choose to do so. Control therapists will provide therapy goals to be used for the COPM-P ratings but will not be instructed to use the COPM or given any other goal-setting tools or strategies.

### Secondary outcomes

**Fig. 4:**
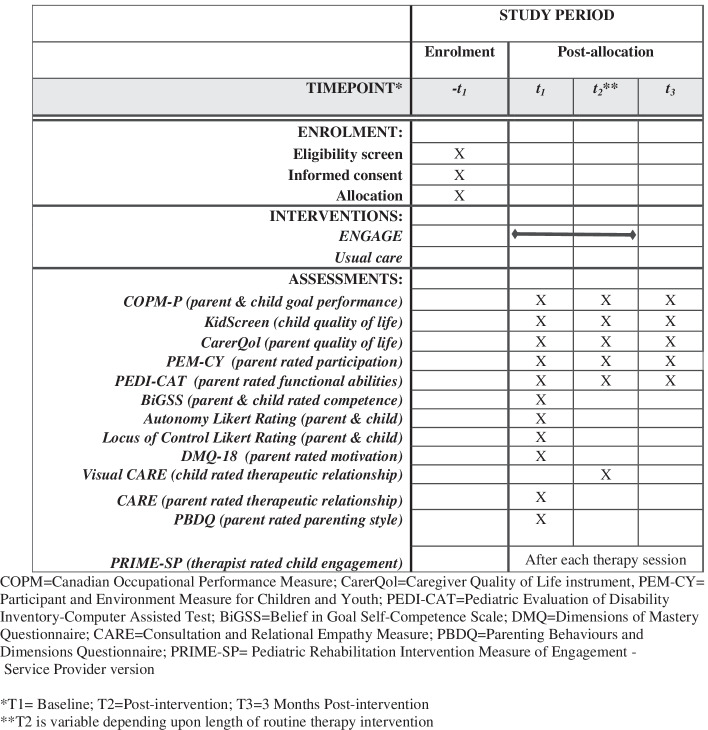
SPIRIT flow chart for study enrolment, interventions and assessments

Secondary outcomes are functional abilities [37], child and parent quality of life [38,39], child engagement [40], and participation [41]. All outcomes will be measured at baseline, post-intervention, and at the 3-month follow-up, administered by trained assessors masked to group allocation. Potential mediators and moderators of intervention effects will also be measured: parenting style [42], perceived autonomy, perceived competence [43], motivation [44], and therapeutic relationship [45, 46] (Fig. 4). We hypothesize that positive differences in favor of the ENGAGE group will be maintained post-intervention and follow-up across all measures.

### Cost

The cost of ENGAGE will be estimated from: compensation of therapists including training costs; any maintenance or licensing fees, cost of materials and supplies associated with operations. Sources for these prices will be provided by the clinical research team.

### Statistical analysis

Data and demographic characteristics will be described (e.g., means, standard deviations) for both groups. Change scores (post minus pre, follow-up minus post) will be summarized for each outcome, with COPM-P change between baseline and post-intervention as the primary analysis. Other outcomes will be used for secondary analyses. For each change score and outcome, a cluster adjusted t-test [47] will be used to compare the mean change score between groups (ENGAGE, control). A confidence interval will be reported for the difference between group mean scores.

Mixed-effects linear regression models on all outcomes will include group and time as fixed effects, a therapist random effect (to adjust for the clustering), and a child random effect (to adjust for repeated measures on each child). Time will be treated as a categorical variable so that the post and follow-up time points can be compared with baseline assessments. A time by group interaction will also be used to assess the effect of group on outcomes. In addition, mixed-effects multiple linear regression models will be developed for each outcome with the additional variables of site, site by group as an interaction to assess site effect and to explore other theoretically important variables (e.g., sex, cognitive abilities, age, parenting style) as covariates. Variables will be dropped from the model one at a time if the p-value is < 0.05 and they are not needed for model fit. This modeling will allow us to assess the effect of the interventions in the presence of important variables that may not be balanced across groups by cluster randomization (e.g., systematic differences in therapists’ caseloads). Main analyses will be based on as per protocol analysis, as recommended for pragmatic trials [48], and performed by an analyst blinded to group assignment using R [49]. Missing data will be examined and multiple imputation methods will be used. A Data Monitoring Committee is not required as ENGAGE does not involve risk above usual care.

### Cost-effectiveness analysis

The ratio of the difference in mean cost between ENGAGE and standard therapy to the difference in mean effectiveness score per group (KidScreen-10) will be used to estimate an incremental cost-effectiveness ratio from the publicly funded healthcare payer and societal perspectives, if ENGAGE is associated with better effectiveness. A bootstrapped analysis will be conducted for analysis. We will assess the between-group difference in cost and effectiveness, ENGAGE vs control, to parameterise distributions for both the difference in cost and effectiveness. We will undertake sensitivity analyses to assess the robustness of the findings to test variations in assumptions regarding uncertain estimates related to costing.

### Implementation evaluation methodology

A multi-method approach will be used to conduct the implementation evaluation. Qualitative and quantitative data are key to gaining a robust and clear understanding of consequential factors to implementation in healthcare settings. Qualitative data will be collected using participant observation during therapist training and bi-weekly therapist meetings with the research team and semi-structured interviews. Quantitative data will be collected through self-report questionnaires. Interpretive Description [50], which focuses on addressing pragmatic, clinically driven problems, will be used as the methodological framework for the qualitative portion of this implementation evaluation.

### Sample

Participants for the implementation evaluation will include therapists in the ENGAGE group (*n* = 12), managers at each site (*n* = 6), and children and parents in the ENGAGE group (*n* = 24 parent and child dyads). Based on previous experience with similar qualitative research, we anticipate the sample size will be adequate to explore implementation. Recruitment of parent and child dyads will be stratified by center, child age and child diagnosis to ensure sample variability.

### Implementation strategy

Implementation support will include informal and formal discussions about anticipated and actual challenges, and potential mitigation strategies to implementation challenges. These will occur with the implementing therapists at each stage of implementation (pre-, during and post-implementation) through scheduled bi-weekly meetings and also on an as-needed basis, and with managers pre- and post-implementation. The aim of these discussions will be to provide implementation support and create a community of practice among the therapists; a strategy that was effective in a study evaluating the implementation of a goal-setting process in pediatric OT [4]. These discussions will enable regular assessment of the factors influencing implementation as they arise during the process so that evidence-based, theory-driven strategies can be leveraged to enhance intervention uptake and use.

### Data collection

**Fig. 5:**
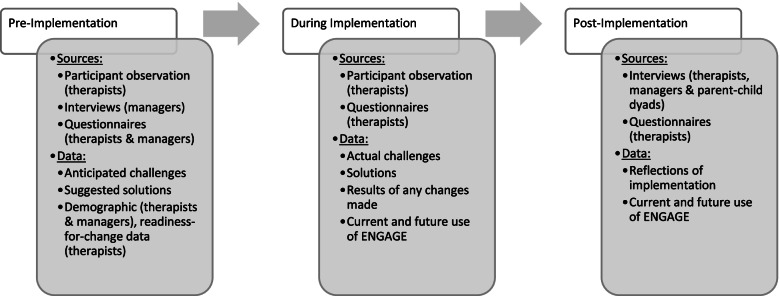
Data collection phases for the implementation evaluation

Data collection will occur in three phases: pre-, during and post-implementation as outlined in Fig. 5. Research team discussions with study therapists related to intervention implementation will be audio-recorded and considered data pre- and during the implementation of ENGAGE. Semi-structured interviews will be conducted pre- (therapists *n* = 12 and managers *n* = 6) and post-implementation (therapists *n* = 12, managers *n* = 6 and parent and child dyads *n* = 24. ENGAGE group therapists will complete two self-report questionnaires: 1) the readiness for change scale of the Organizational Change Questionnaire – Climate of Change, Processes, and Readiness measure [51] (prior to training), and 2) three global questions about use and intended future use of ENGAGE in clinical practice pre- and post- intervention [52, 53]. Therapists and managers will also complete a short demographic questionnaire.

### Data analysis

Inductive and deductive approaches will be used to analyze the qualitative data, which will include transcripts from each phase of data collection, study notes, and meeting syntheses. A deductive approach will be conducted to provide a specific implementation lens on barriers, facilitators and contextual influences of implementation using the Consolidated Framework for Implementation Research (CFIR) [24] and Theoretical Domains Framework (TDF) [54] combined [55]. Transcripts will be analyzed line by line independently by two researchers to identify text that directly maps to the constructs in the combined framework. The purpose of this approach to analysis will be to rigidly apply the qualitative data to the combined framework. Discrepancies will be resolved through discussion. Codes will be organized in table format as frequency counts in the domains of CFIR and TDF for all rehabilitation sites. To compare differences across rural and urban rehabilitation sites, a table will also be created for codes separated by rural and urban locations.

The inductive approach to data analysis will provide rich contextual details on the therapists, managers and families' experiences around the implementation process that will not be captured in the deductive analysis. The inductive analysis will be informed by Braun & Clarke's 6-phase framework [56].

### Rigor

*Journaling –* Journaling will be used to record data collection, analysis or general methodological reflections and decisions. Following each interview, the interviewer will record where the interview took place and any relevant contextual details that would facilitate the analysis. Preliminary ideas about the data will also be noted shortly following the interview and will inform subsequent analysis.

*Peer/Mentor Debrief –* The analysis will be conducted by a minimum of two members of the research team. The transcripts will be reviewed and coded independently, and then discussed at meetings. The purpose of these ongoing interactions is to ensure regular critical discussions and reflections on methodological decisions and interpretations during data collection and analysis.

## Discussion

While previous research has evaluated the effectiveness of goal-setting processes embedded in other intervention strategies [32, 57], this will be the first study to evaluate the effectiveness of a child-focused goal-setting approach on child outcomes as reported by parents and children. The concurrent implementation evaluation and cost-effectiveness analyses will provide valuable information for implementation in a wide range of pediatric rehabilitation settings. Overall, attempted formalized goal-setting processes are inconsistently implemented in pediatric rehabilitation [11, 58]. Implementation evaluations are used to identify and understand barriers and facilitators to implementation and to identify successful strategies that can be used to optimize intervention uptake and use. We will use an evidence-based and theory-driven approach to implementation within this trial.

Robust theoretical frameworks of goal-related performance and behavior change are also underutilized in pediatric rehabilitation. The evidence base is mainly atheoretical and focused on clinical issues related to implementing specific goal-setting tools [13]. This gap is notable as there are relevant theoretical frameworks and related evidence in other fields that would enhance and inform practice and research in pediatric rehabilitation. For example, self-determination theory purports three precursors for motivation: autonomy, relatedness and competence [59]. Goal-setting theory [14] suggests that goal-related performance is enhanced with goals that are specific, challenging, proximal and important to the individual whose behavior is expected to change. Social cognitive theory [60] has been supported among adults receiving rehabilitation who demonstrate a relationship between perceived self-efficacy and performance [61–63]. It is likely that collaborative goal-setting processes that facilitate child autonomy, relatedness, and competence would increase child motivation to participate in goal-related therapy, and thus improve child outcomes [16, 64, 65], however evidence is needed to confirm these relationships in the pediatric rehabilitation setting. An in-depth understanding of the barriers and facilitators to the implementation of ENGAGE, as well as possible mitigation strategies, will enhance future uptake and ultimately widespread implementation of this goal-setting approach. Any plans to change this protocol will be communicated via the clinical trials registry and communicated with participants as per Ethics Board requirements.

## Data Availability

The datasets used and/or analysed during the current study will be available from the corresponding author on reasonable request and approval of the Health Research Ethics Board at the University of Alberta.

## Abbreviations

OT: Occupational Therapy
PT: Physical Therapy
ENGAGE: Enhancing Child Engagement in Goal-Setting
RCT: Randomized Controlled Trial
COPM: Canadian Occupational Performance Measure
ICC: Intra-Cluster Correlation
REDCap: Research Electronic Data Capture
CONSORT: Consolidated Standards of Reporting Trials
PEGS: Perceived Efficacy and Goal Settin
CarerQol: Caregiver Quality of Life
PEM-CY: Participant and Environment Measure for Children and Youth
PEDI-CAT: Pediatric Evaluation of Disability Inventory-Computer Assisted Test
BiGSS: Belief in Goal Self-Competence Scale
DMQ: Dimensions of Mastery Questionnaire
CARE: CARE Patient Feedback Measure
PBDQ: Parenting Behaviours and Dimensions Questionnaire
PRIME-SP: Pediatric Rehabilitation Intervention Measure of Engagement – Service Provider version
ICER: Incremental Cost-Effectiveness Ratio
CFIR: Consolidated Framework for Implementation Research
TDF: Theoretical Domains Framework
